# Prevalence of basilar artery variants: a systematic review with meta-analysis of radiological studies

**DOI:** 10.1007/s00234-026-04008-6

**Published:** 2026-04-29

**Authors:** George Triantafyllou, Ioannis Paschopoulos, Panagiotis Papadopoulos-Manolarakis, Nikolaos-Achilleas Arkoudis, Georgios Velonakis, Panagiotis Papanagiotou, Maria Piagkou

**Affiliations:** 1 Department of Anatomy, School of Medicine, Faculty of Health Sciences, National and Kapodistrian University of Athens, Athens, Greece; 2 Department of Neurosurgery, General Hospital of Nikaia-Piraeus, Athens, Greece; 3 Research Unit of Radiology and Medical Imaging, National and Kapodistrian University of Athens, Athens, Greece; 4 Second Department of Radiology, General University Hospital “Attikon”, National and Kapodistrian University of Athens, Athens, Greece; 5 Department of Radiology, Aretaieion University Hospital, School of Medicine, National and Kapodistrian University of Athens, Athens, Greece

**Keywords:** Basilar artery, Variation, Neuroradiology, Evidence-based anatomy, Meta-analysis

## Abstract

**Purposes:**

To provide a comprehensive meta-analytic synthesis of the morphological variability of the basilar artery (BA) - specifically fenestrations, persistent trigeminal arteries (PTA), and aberrant posterior inferior cerebellar artery (PICA) origin - and to elucidate their clinical implications for neuroradiological and neurosurgical procedures.

**Methods:**

Following PRISMA 2020 and Evidence-Based Anatomy guidelines, a systematic literature search of three databases was performed. Inclusion criteria focused on imaging studies (CTA, MRA, DSA) reporting the prevalence of specific BA variants. Pooled prevalence was calculated using random-effects models via R programming software.

**Results:**

Thirty-five studies involving 266,696 patients were included. The aberrant PICA origin from the BA was the most frequent variant, with a pooled prevalence of 4.53% (95% CI: 0.06–14.52). BA fenestration followed with a prevalence of 1.36% (95% CI: 0.93–1.86), showing a statistically significant difference in detection between MRA (1.69%) and DSA (0.24%). The rarest variant was the PTA, calculated at 0.20% (95% CI: 0.11–0.31). Considerable heterogeneity was observed across pooled estimates.

**Conclusions:**

Although BA variations are relatively infrequent, their precise identification is a clinical necessity for procedural safety. Awareness of these variants is vital for navigating complex vascular geometries and managing the potential risk of associated aneurysms during endovascular interventions.

**Supplementary Information:**

The online version contains supplementary material available at 10.1007/s00234-026-04008-6.

## Introduction

The anatomy of the intracranial arterial circulation has been re-examined extensively in recent years, largely due to the routine use of high-resolution vascular imaging. Computed tomography angiography (CTA), magnetic resonance imaging (MRA), and digital subtraction angiography (DSA) now permit detailed visualization of the posterior circulation in large patient cohorts [1–3].

Typically, the vertebrobasilar system (VBS) supplies the posterior circulation of the brain. The vertebral arteries (VA) unite at the pontomedullary junction to form the basilar artery (BA), which courses along the ventral pons before dividing into the posterior cerebral arteries. In general, the BA provides essential arterial supply to the brainstem (via perforating-pontine arteries), cerebellum (via the anteroinferior and superior cerebellar arteries), and posterior cerebral hemispheres (via the posterior cerebral arteries) [4].

The VBS has substantial morphological variability, including aberrant origins [5], aberrant branches [6], fenestrations [7] and persistence of primitive interconnections with the internal carotid artery (ICA) system [8].

Although several evidence-based meta-analyses were performed for the cerebral arterial circle [5, 7, 9–11], to the authors’ knowledge, there are no meta-analytic evidence for the BA variations. Published reports remain heterogeneous with respect to imaging modality, study population, and variant definitions, which likely explains the wide range of reported prevalence values. Thus, the purpose of the current paper is to review the morphological variability of the BA and link it with their clinical implications, especially for neuroradiological and neurosurgical procedures.

## Materials and methods

The systematic review with meta-analysis adhered to the guidelines proposed by the Evidence-Based Anatomy Workgroup for anatomical meta-analysis [12] and the PRISMA 2020 statement for systematic reviews [13]. The protocol was not registered in PROSPERO, due to the suspension of the database for prevalence meta-analyses.

A comprehensive literature review was conducted using the online databases PubMed, Scopus, and Web of Science, from their respective inception dates until January 2026. The following terms were employed in various combinations: “basilar artery,” “variation,” “basilar artery fenestration,” “persistent trigeminal artery,” “posterior inferior cerebellar artery,” “imaging study,” and “radiological study”. Boolean operators (AND, OR) and database-specific field tags were applied to optimize retrieval sensitivity. An example of the search strategy is provided in Table 1. Additionally, the references of all included articles were assessed, and a thorough search of key anatomical and neuroradiological journals (Neuroradiology, American Journal of Neuroradiology, The Neuroradiology Journal, Journal of Neuroradiology, Interventional Neuroradiology, Clinical Neuroradiology, Annals of Anatomy, Clinical Anatomy, Journal of Anatomy, Anatomical Record, Surgical and Radiological Anatomy, Folia Morphologica, European Journal of Anatomy, Morphologie, Anatomical Science International, Anatomy and Cell Biology) was conducted. There were no date or language restrictions. The inclusion criteria comprised studies that reported the prevalence of BA variations via imaging studies. The following variants were recorded in the current literature: (i) BA fenestration, (ii) presence of persistent trigeminal artery (PTA) interconnecting the BA with the internal carotid system, and (iii) aberrant posterior inferior cerebellar artery (PICA) originating from the BA. Cadaveric studies, studies including only pathological population, case reports, conference abstracts, animal studies, and studies presenting irrelevant or insufficient data were excluded.

**Table 1:** Example of the search combinations used in the current systematic review with meta-analysis

Search number	Search term combinations
1	((basilar artery [Title/Abstract]) AND (variation [Title/Abstract])) AND ((imaging study [Title/Abstract]) OR (radiological study [Title/Abstract]))
2	(basilar artery fenestration [Title/Abstract]) AND ((imaging study [Title/Abstract]) OR (radiological study [Title/Abstract]))
3	(persistent trigeminal artery [Title/Abstract]) AND ((imaging study [Title/Abstract]) OR (radiological study [Title/Abstract]))
4	((posterior inferior cerebellar artery [Title/Abstract]) AND (variation [Title/Abstract])) AND ((imaging study [Title/Abstract]) OR (radiological study [Title/Abstract]))

Two independent reviewers (GT, IP) screened titles, abstracts, and full texts and extracted the data into standardized Microsoft Excel sheets. Discrepancies were resolved by consensus following discussion with the senior authors. Extracted variables included year of publication, country, imaging modality, sample size, and number of cases per variant. The Anatomical Quality Assurance (AQUA) tool, developed by the Evidence-Based Anatomy Workgroup for anatomical reviews, was used to assess each article’s risk of bias [14].

A statistical meta-analysis was performed using the open-source R programming language and RStudio software (version 4.3.2), with the “meta” and “metafor” packages by a single investigator (GT). The pooled prevalence was calculated using inverse variance and random effects models. The proportions (prevalence) meta-analysis was conducted using the Freeman-Tukey double arcsine transformation, the DerSimonian-Laird estimator for the between-study variance tau², and the Jackson method for the confidence interval of tau² and tau. Furthermore, several subgroup analyses were conducted to identify variables (geographic distribution or imaging technique) affecting the estimated pooled prevalence. A p-value of less than 0.05 was deemed statistically significant. Cochran’s Q statistic was utilized to assess heterogeneity across studies (*p* < 0.10 considered significant), while the Higgins I² statistic quantified the degree of inconsistency. I² values between 0 and 40% were regarded as low, 30–60% as moderate, 50–90% as substantial, and > 75% as considerable heterogeneity. To evaluate the robustness of the pooled prevalence estimates and identify potential sources of heterogeneity, a leave-one-out sensitivity analysis was performed. This procedure involved systematically omitting one study at a time and recalculating the pooled effect size and the Higgins I2 statistic to determine if any single dataset disproportionately influenced the overall results. To investigate the small-study effect (the phenomenon where smaller studies may exhibit different effects than larger ones), the DOI plot with the LFK index was generated [15].

## Results

### Search analysis

**Fig. 1:**
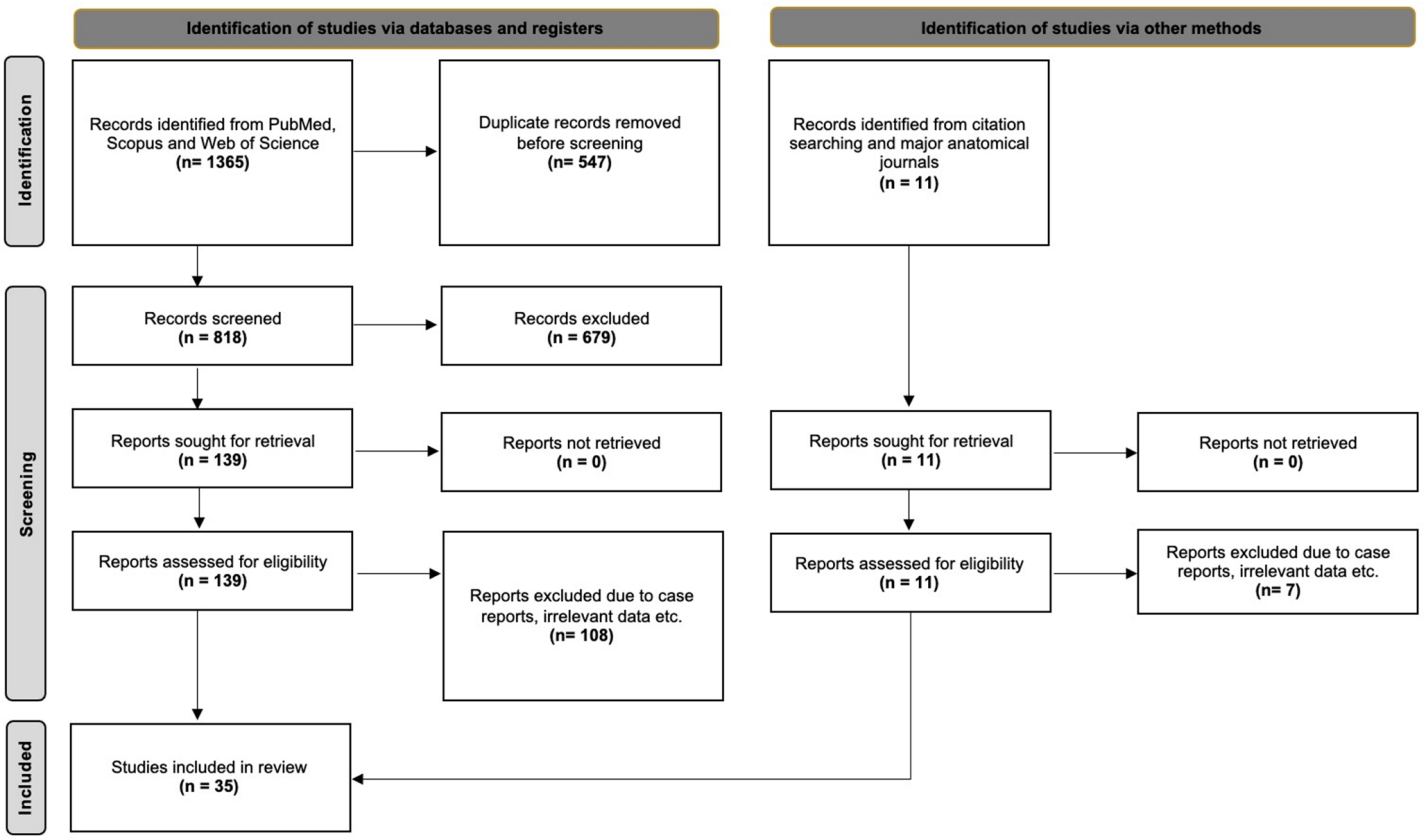
The search analysis flow chart according to the PRISMA 2020 guidelines

The database search yielded 1,365 articles exported to Mendeley version 2.10.9 (Elsevier, London). After excluding duplicates and screening titles and abstracts for eligibility, 139 studies were retrieved and screened for full text. Ultimately, 31 studies were deemed eligible for inclusion in the systematic review. Additionally, four studies were identified through manual reference screening and targeted journal searches. Hence, 35 studies were included in our systematic review with meta-analysis. Figure 1 summarizes the flow diagram of our search analysis, following the PRISMA 2020 guidelines [13].

### Eligible characteristics of eligible studies

Thirty-five (35) studies were included, comprising 266,696 patients. The mean sample size per article was 7,619.89 patients. Concerning the imaging technique, fifteen (15) studies were conducted using MRA, eleven (11) studies were based on CTA scans, and four utilized angiograms. The remaining five studies used a combination of imaging modalities. Concerning the population, twenty-two (20) studies had Asian population, nine (9) with the European population and four (4) with American. The characteristics of the included studies, including details of the imaging techniques, are summarized in Table 2.

**Table 2:** The characteristics of the eligible studies, including their risk of bias assessment based on the AQUA tool (Henry et al., 2017 [14])

Study	Year	Population	Type	Patients	Bias
Akgun et al. [16]	2013	Turkey	CTA (64-slices, 0.5 mm slice thickness) - MRA (3T)	188	Low
Alharbi et al. [17]	2024	Saudi Arabia	MRA (1.5T)	85	Low
Allen et al. [18]	2005	USA	DSA	481	Low
Arraez-Aybar et al. [19]	2013	Spain	MRA (1.5T)	200	Low
Arraez-Aybar et al. [20]	2015	Spain	CTA (64-slices, 0.5 mm slice thickness) - MRA (1.5T) - DSA	169	High
Bai et al. [21]	2013	China	MRA (3T)	6095	Low
Bayrak et al. [22]	2011	Turkey	CTA (64-slices, 0.9 mm slice thickness)	395	Low
Bharatha et al. [23]	2008	Canada	CTA (64-slices)	504	High
Bozek et al. [24]	2012	Poland	CTA	1140	Low
Bulbuc et al. [25]	2025	Romania	MRA (1.5T)	860	Low
Chen et al. [26]	2011	China	MRA (3T)	4650	Low
Czyzewski et al. [27]	2022	Poland	CTA (64- / 256-slices, 0.5 mm slice thickness)	6545	Low
Deniz et al. [28]	2022	Turkey	CTA (64-slices, 0.8 mm slice thickness)	1150	Low
Dodevski et al. [29]	2011	North Macedonia	CTA	50	High
Hamidi et al. [30]	2013	Turkey	CTA (64-slices, 0.9 mm slice thickness)	500	Low
Huang et al. [31]	2023	China	CTA (64-slices, 0.75 slice thickness) - MRA (1.5T)	94,487	Low
Kim and Kim. [32]	2015	South Korea	MRA (1.5T) - CTA (64-slices, 0.5 mm slice thickness) - DSA	8900	Low
Kovac et al. [33]	2014	Serbia	CTA (16-slices, 0.625 slice thickness)	455	High
Liu et al. [34]	2014	China	MRA (1.5-3T)	48,184	Low
Mei et al. [35]	2025	China	MRA (3T)	9986	Low
Melissanidis et al. [36]	2025	Greece	CTA (128-slices)	200	Low
O’uchi and O’uchi [37]	2010	Japan	MRA (1.5T)	16,415	Low
Park et al. [38]	1998	Korea	DSA	3353	High
Pekcevik and Pekcevik [39]	2014	Turkey	CTA (64-slices, 0.5 mm slice thickness)	341	Low
Sanders et al. [40]	1993	USA	DSA	5190	Low
Siqueira et al. [41]	1992	Brazil	DSA	5500	High
Sogawa et al. [42]	2013	Japan	MRA (1.5T)	16,471	Low
Triantafyllou et al. [43]	2025	Greece	CTA (128-slices, 0.6–0.8 mm slice thickness)	500	Low
Uchino et al. [44]	2000	Japan	MRA (1.5T)	523	Low
Uchino et al. [45]	2001	Japan	MRA (1.5T)	600	Low
Uchino et al. [46]	2012	Japan	MRA (1.5T)	3327	Low
Uchino et al. [47]	2012	Japan	MRA (1.5T)	3491	Low
Uchino et al. [48]	2014	Japan	MRA (1.5-3T)	72	High
Weon et al. [49]	2011	Korea	MRA (1.5-3T)	7329	Low
Wu et al. [50]	2020	China	CTA (320-slices, 1.0 mm slice thickness) and MRA (3T)	18,360	Low

### Meta-analysis outcomes

**Fig. 2:**
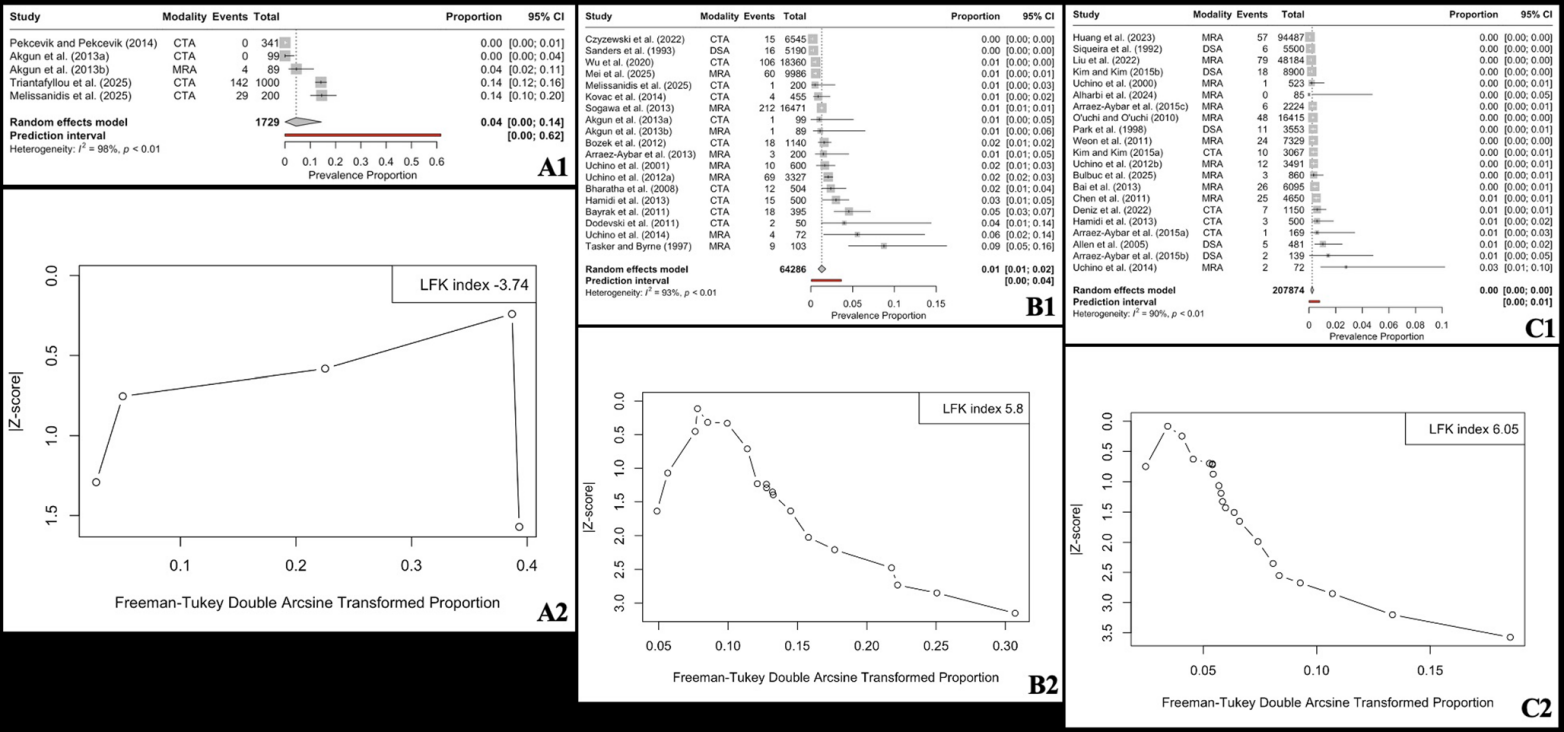
Forest (**A1**, **B1**, **C1**) and DOI (**A2**, **B2**, **C2**) plots for the estimated pooled prevalence of posteroinferior cerebellar artery (PICA) originating from the basilar artery (BA) (**A1**, **A2**), BA fenestration (**B1**, **B2**) and persistent trigeminal artery (PTA) presence (**C1**, **C2**)

**Fig. 3:**
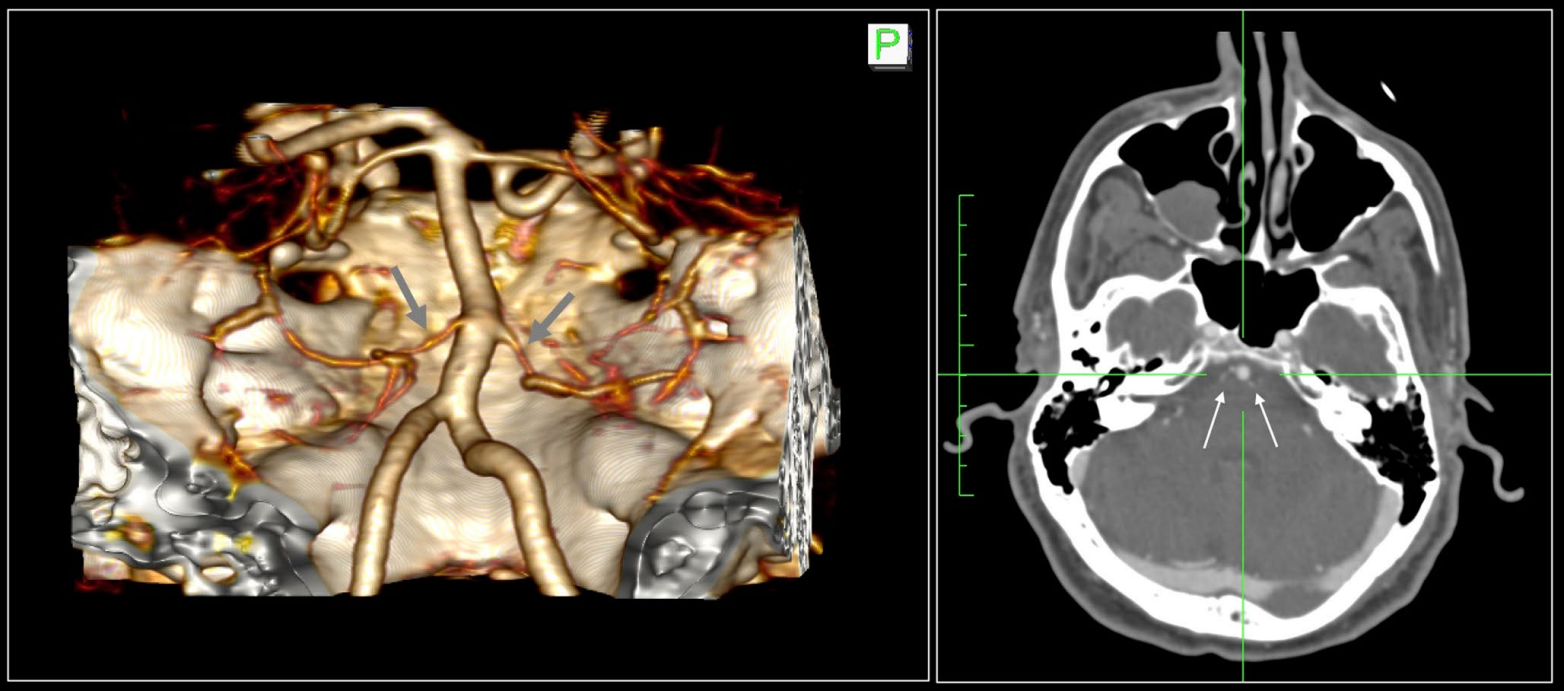
Computed tomography angiography (CTA) with 0.8 mm slice thickness. Three-dimensional volume and axial reconstructions of bilateral posteroinferior cerebellar artery (PICA) originating from the basilar artery (BA)

An aberrant PICA originating from the BA was the most common variation observed with a pooled prevalence of 4.53% (95% CI: 0.06–14.52) (Figs. 2 and 3). The Higgins I² was 97.6%, indicating considerable heterogeneity. The DOI plot had a LFK index of -3.74 (major asymmetry), indicating potential small-study effect (Fig. 2). Imaging technique subgroup analysis was statistically insignificant (*p* = 0.8273). Leave-one-out sensitivity analysis pinpointed the study by Triantafyllou et al. [43] as a major statistical driver. Removing this single study, which reported an individual 14.2% prevalence [43], caused a notable 2.66% drop in the overall pooled estimate (shifted Higgins I² = 95.9%) (Supplementary Fig. 1). This suggests that the high heterogeneity is a reflection of the contrast between modern high-resolution imaging series [43] and older-smaller cohorts [39].

**Fig. 4:**
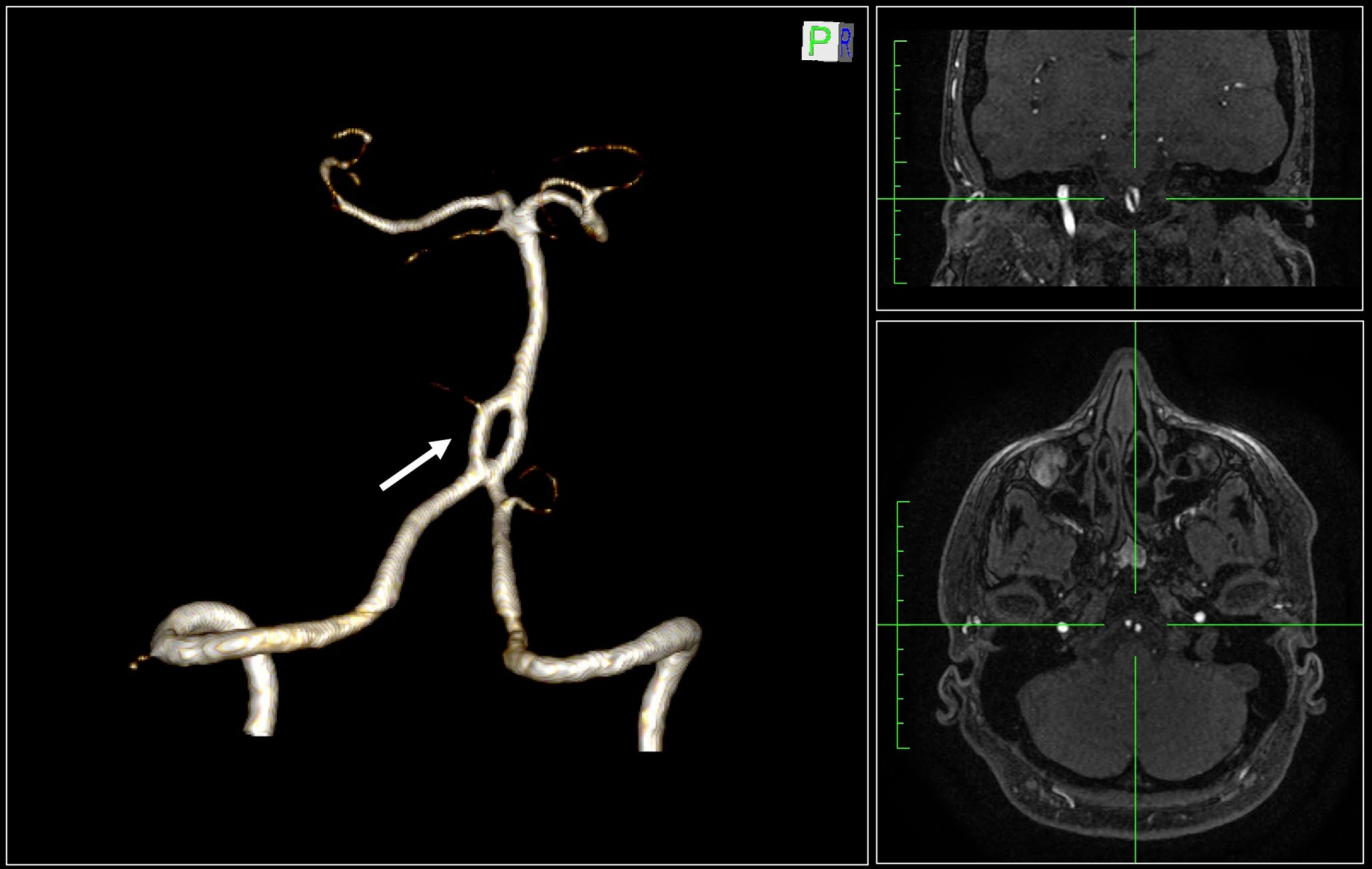
Magnetic resonance angiography (MRA) of 1.5 Tesla with time-of-flight (TOF) modality. Three-dimensional volume, axial and coronal reconstructions of basilar artery (BA) fenestration

The second most frequent variation was the BA fenestration, and it was estimated with a pooled prevalence of 1.36% (95% CI: 0.93–1.86) (Figs. 2 and 4). The Higgins I² was 93.1%, indicating considerable heterogeneity. The DOI plot had a LFK index of + 5.8, depicting major asymmetry and possible small-study effect (Fig. 2). The nationality subgroup analysis did not depict a statistically significant result (*p* = 0.8866). The imaging technique subgroup analysis demonstrated a statistically significant difference (*p* < 0.0001), with DSA studies having the lowest prevalence (0.24%) and MRA studies the highest (1.69%). Regarding the site of fenestration, the proximal BA was the most common location with a pooled prevalence of 1.21% (95% CI: 0.69–1.86), followed by the vertebrobasilar junction [0.11% (95% CI: 0.00-0.58)] and the distal BA [0.04% (95% CI: 0.00-0.24). The leave-one-out sensitivity analysis did not identify significant results with no outlier studies (Supplementary Fig. 2). Thus, the prevalence estimate is confirmed between large-scale MRA datasets with a reliable baseline for this morphological feature [18, 19].

**Fig. 5:**
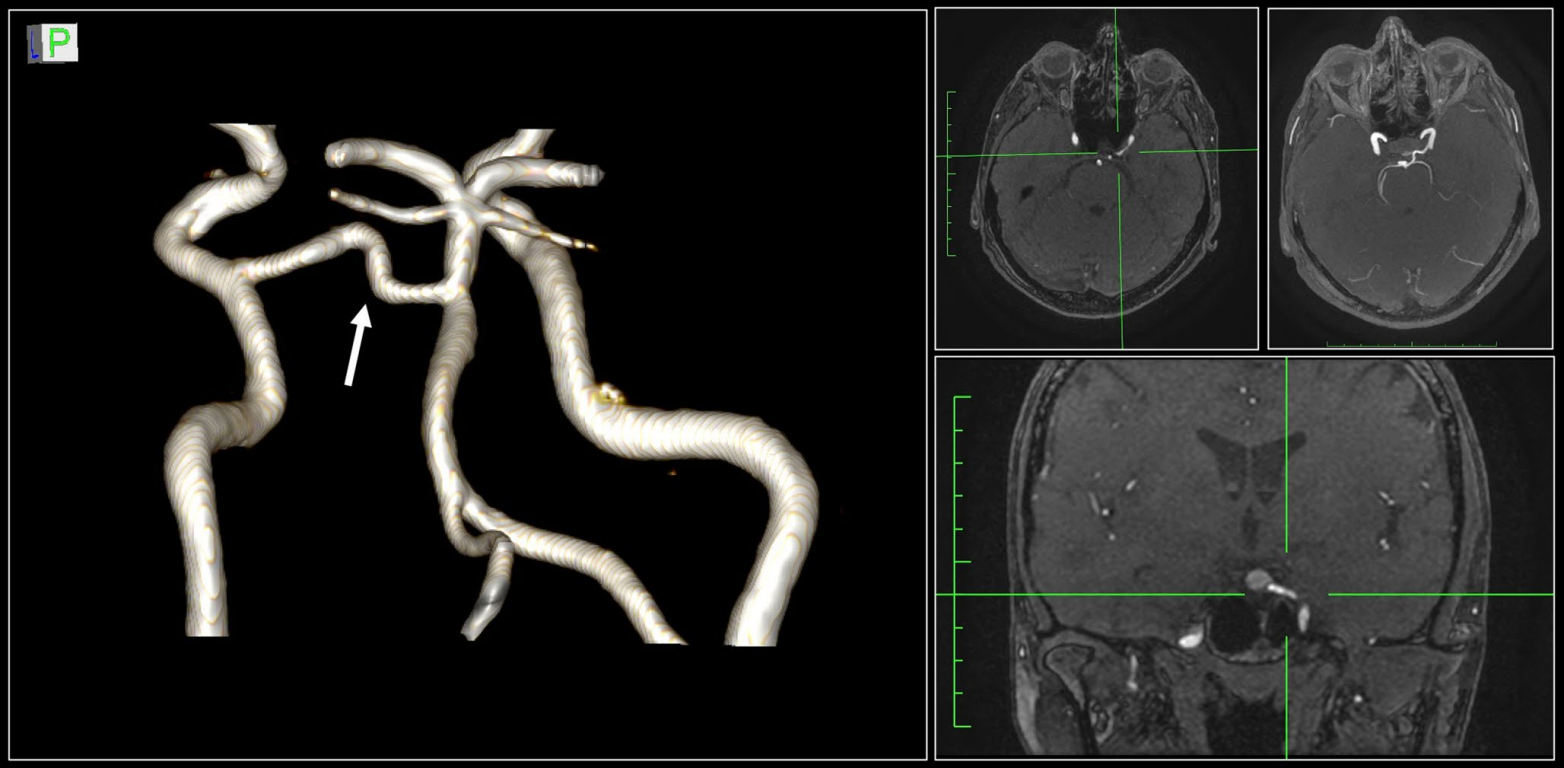
Magnetic resonance angiography (MRA) of 1.5 Tesla with time-of-flight (TOF) modality. Three-dimensional volume, axial and coronal reconstructions of persistent trigeminal artery (PTA) interconnecting the basilar artery (BA) and the internal carotid artery (ICA)

The rarest variant was the presence of PTA, and it was calculated with a pooled prevalence of 0.20% (95% CI: 0.11–0.31) (Figs. 2 and 5). The Higgins I² was 90.0%, indicating substantial heterogeneity. The DOI plot had a LFK index of + 2.19 (major asymmetry), indicating possible small-study effect (Fig. 2). The nationality and imaging technique subgroup analysis did not show a statistically significant difference (*p* = 0.7475 and *p* = 0.3791, respectively). Side difference was also statistically insignificant (*p* = 0.6773). The statistical weight for this variant was heavily dominated by the study of Huang et al. [31]. Leave-one-out sensitivity analysis demonstrated that excluding this specific study did not alter the pooled prevalence estimate, but caused a significant drop to the heterogeneity, with shifter Higgins I² = 72.9% (Supplementary Fig. 3).

## Discussion

The current systematic review with meta-analysis provides a large-scale quantitative synthesis of the BA variants according to 35 studies and 266,696 patients. Along with the previous meta-analysis of the VA first-second [5] and third-fourth segments [7], the present work contributes to completing the evidence-based anatomical assessment of the VBS. Our results highlight that the variations of the BA are infrequent, with the aberrant PICA origin being the most common (4.53%). However, given the considerable heterogeneity observed across studies, these pooled estimates should be interpreted as aggregated averages rather than fixed epidemiological constants. Between the radiological studies, all the imaging techniques (CTA, MRA, DSA) were sensitive in identifying the variations; however, statistically significant difference was identified for BA fenestration. The anatomical and clinical considerations of the current results will be further discussed, especially for their implications in diagnostics and interventional procedures.

### Aberrant basilar origin of posteroinferior cerebellar artery

The results of our systematic review and meta-analysis identify an aberrant PICA origin from the BA as the most frequent variation, with a pooled prevalence of 4.53%. While this variant showed considerable heterogeneity, it was consistently detectable across different imaging modalities, as the subgroup analysis for imaging techniques remained statistically insignificant. In our primary radiological cohort, the BA was identified as the third most common source of the PICA, appearing in 14.2% of the 1,000 sides evaluated [43]. This proportion exceeds the pooled estimate of the present meta-analysis and likely reflects population-specific factors or differences in imaging resolution and classification criteria. High-resolution CTA series have reported higher detection rates than traditional cadaveric studies [43, 51]. In our proposed classification system, this is designated as Type 3 PICA origin [43]. Bilateral PICA origin from BA were relatively uncommon, documented in only 3.2% of patients [43]. It is important to note that the PICA territory typically includes the dorsolateral medulla, inferior cerebellar hemisphere, and vermis. Consequently, occlusion or iatrogenic injury to a BA-origin PICA can lead to lateral medullary (Wallenberg) syndrome or debilitating cerebellar infarction [51].

From an interventional neuroradiology perspective, recognizing a BA origin is essential for safe procedural execution. Sharp angulations at the junction often complicate microcatheter navigation, and the small size of these vessels increases the risk of rupture or coil migration during treatment [52]. While PICA aneurysms are generally rare, representing only 0.5–3% of all intracranial aneurysms, their management is significantly influenced by the parent vessel origin [52]. In proximal or ostial configurations, flow diversion has emerged as a technically feasible option with a reported complete occlusion rate of 81%, while it carries a notable complication rate of 18% [53]. Preoperative identification via CTA or DSA is vital, as the specific origin and course of the artery may dictate whether endovascular or open surgical approaches are feasible [53].

### Basilar artery fenestration

The second most frequent variation identified in our meta-analysis was BA fenestration, which was estimated to have a pooled prevalence of 1.36%. The imaging technique used was a statistically significant factor (*p* < 0.0001). Specifically, DSA studies reported the lowest prevalence (0.24%), whereas MRA studies showed the highest (1.69%). This discrepancy likely reflects technical limitations of planar angiography compared with high-resolution three-dimensional reconstructions available with CTA and MRA. Anatomically, BA fenestration represents a duplication of a segment of the artery, resulting in two distinct channels that subsequently reunite [46]. This variation arises from the incomplete fusion of the primitive longitudinal neural arteries during early embryonic development [46].

Clinically, while often asymptomatic and found incidentally, BA fenestration is of significant importance because it has been associated with a higher incidence of intracranial aneurysms, particularly at the proximal end of the fenestrated segment [54–56]. The altered hemodynamics and structural weaknesses at the points of bifurcation and reunion within the fenestration are thought to predispose these sites to aneurysm formation [54–56]. Furthermore, the presence of a fenestration can complicate endovascular procedures, as the dual-lumen anatomy may affect catheter navigation and the deployment of stents or flow diverters [56]. In interventional planning, precise mapping of the fenestration is crucial, as the small caliber of the fenestrated limbs increases their vulnerability to iatrogenic injury [54]. High-resolution imaging, such as CTA or MRA, is often necessary to distinguish a true fenestration from other pathologies like arterial dissection or a duplicated artery [54–56]. For patients undergoing treatment for aneurysms associated with BA fenestration, the choice between endovascular coiling, clipping, or flow diversion must be carefully individualized based on the specific morphology of the fenestration and the aneurysm’s relationship to the bifurcated segments [54–56].

### Persistent trigeminal artery

The rarest variation documented in our systematic review was the presence of PTA, which was calculated to have a pooled prevalence of 0.20%. Subgroup analyses for nationality, imaging technique, and side difference did not yield statistically significant results. The PTA represents the most common persistent embryonic carotid-basilar anastomosis, serving as a direct channel between the ICA and the BA [8]. Anatomically, the PTA typically originates from the cavernous segment of the ICA and joins the BA between the origins of the superior cerebellar and the anterior inferior cerebellar arteries [8]. In some cases, the PTA may terminate directly in a cerebellar artery, such as the PICA, rather than joining the main trunk of the BA. These configurations are called PTA variants, and they were not included in the current review because they do not represent BA variations [57]. Clinically, the presence of a PTA is significant because it is often associated with hypoplasia of the proximal vertebrobasilar system, as the PTA becomes the primary source of blood flow to the posterior circulation [57]. This configuration can lead to complex hemodynamic shifts and an increased risk of ischemic events if the carotid system is compromised [57].

From a surgical and interventional perspective, the identification of a PTA is vital for safety during neurovascular procedures. The artery often has a close relationship with the abducens nerve and other structures within the cavernous sinus, making it vulnerable during skull base surgeries [57–59]. Additionally, PTA must be recognized during endovascular planning for aneurysms or mechanical thrombectomy, as the anomalous connection dictates the route of catheter access and the potential for collateral flow [58, 60]. Understanding these categories are essential to avoid inadvertent injury and to anticipate the specific requirements for catheter support and dural crossing [57–60].

### Rare variations of basilar artery

In the current literature, isolated case reports have described rarer BA variants. Similar to BA fenestrations, embryological alterations during the fusion of the primitive longitudinal arteries can lead partial (proximal or distal) or complete duplication of the BA [61]. These variants have been demonstrated in Uchino [61] textbook and in few case reports [62]. Another similarly derived embryologically variation is the double fenestration of the BA, also reported in sporadic cases [61, 63]. Furthermore, recently, we reported the primitive lateral vertebrobasilar anastomosis – a rare variation (0.4% estimated prevalence) that should be clearly differentiated from BA fenestration at the junction with the VA [64].

Exceedingly rare, aberrant branches can be seen arising from the BA – except from the PICA. Kumar and Mishra [65] recorded the origin of the middle meningeal artery from the distal BA with the use of DSA. Sade et al. [66] identified the ophthalmic artery originating from the BA with DSA technique, while Bhogal et al. [67] observed an orbital branch arising from the BA that supplied the extra-ocular muscles. These variants are exceedingly uncommon but underscore the embryological complexity of VBS development.

### Topographical associations of basilar artery

The topographical relationships of the BA are of paramount importance in the neuroanatomical assessment of the posterior fossa, as they dictate the surgical approach and endovascular strategy for complex lesions. Neuroradiological imaging, particularly CTA and MRA, is essential for mapping the BA trajectory relative to the surrounding structures. The relationship between the BA and the fourth ventricle is vital, as distal branches like the PICA supply the choroid plexus and the roof of the ventricle, while the artery should be carefully mapped during ventriculostomy procedures [68]. From a neuroradiological standpoint, the height of the BA tip relative to the posterior clinoid processes is a critical prognostic factor for microsurgical and endovascular aneurysm treatment [69]. A ‘high’ BA tip often facilitates endovascular access but may alter the hemodynamics of the terminal branches [69]. Conversely, a ‘low’ bifurcation can be hidden by the bony anatomy of the sella, complicating the placement of flow diverters or stents [69].

### Limitations

Several limitations must be acknowledged. All pooled estimates demonstrated high heterogeneity (I² > 90%), likely reflecting differences in imaging resolution, population demographics, and variant definitions. Our leave-one-out analysis further illustrated that the pooled prevalence values are highly sensitive to individual studies with extreme results [43] or massive sample sizes [31]. However, the shifted I^2^ results during the analysis remained considerable to substantial. Therefore, pooled prevalence values should be interpreted cautiously and not assumed to represent uniform global rates. DOI plots suggested potential small-study effects for the prevalence estimates. Additionally, retrospective study designs predominated among the included studies, increasing the risk of selection bias. Differences in sensitivity among CTA, MRA, and DSA may also have influenced detection rates of small-caliber variants.

## Conclusions

The current systematic review and meta-analysis provide a comprehensive analysis of BA variations based on 266,696 patients. Our findings establish that an aberrant origin of the PICA from the BA is the most prevalent variation, occurring with a pooled prevalence of 4.53%. BA fenestration follows as the second most common variant at 1.36%, with its detection significantly influenced by the choice of imaging modality, favoring high-resolution three-dimensional techniques like MRA over traditional DSA. The PTA remains the rarest of the variants, identified in only 0.20% of the population. From a neuroradiological and interventional perspective, the precise identification of these variations is a clinical necessity for procedural safety. Knowledge of an aberrant PICA origin or a proximal BA fenestration is vital for navigating complex vascular geometries and managing the increased risk of associated aneurysms. Recognizing the presence of persistent embryonic vessels, like the PTA, allows for better anticipation of hemodynamic shifts and collateral flow requirements during endovascular interventions. These results offer a standardized framework that can enhance diagnostic accuracy and assist in the individualized planning of neurosurgical and endovascular treatments within the posterior fossa. Future prospective, standardized imaging studies are warranted to refine prevalence estimates and reduce heterogeneity across populations.

## Supplementary information

Below is the link to the electronic supplementary material.

Supplementary Material 1

## Data Availability

The data are available upon reasonable request to the corresponding author.
