# Supplementary material for: Prevalence of basilar artery variants: a systematic review with meta-analysis of radiological studies
Source: Neuroradiology. 2026 Apr 29;68(6):1607–17. doi: 10.1007/s00234-026-04008-6 (PMC13323284; doi:10.1007/s00234-026-04008-6)
Supplement: Supplementary file 1 — Supplementary Material 1 [file 234_2026_4008_MOESM1_ESM.docx]

**Supplementary Materials**

**
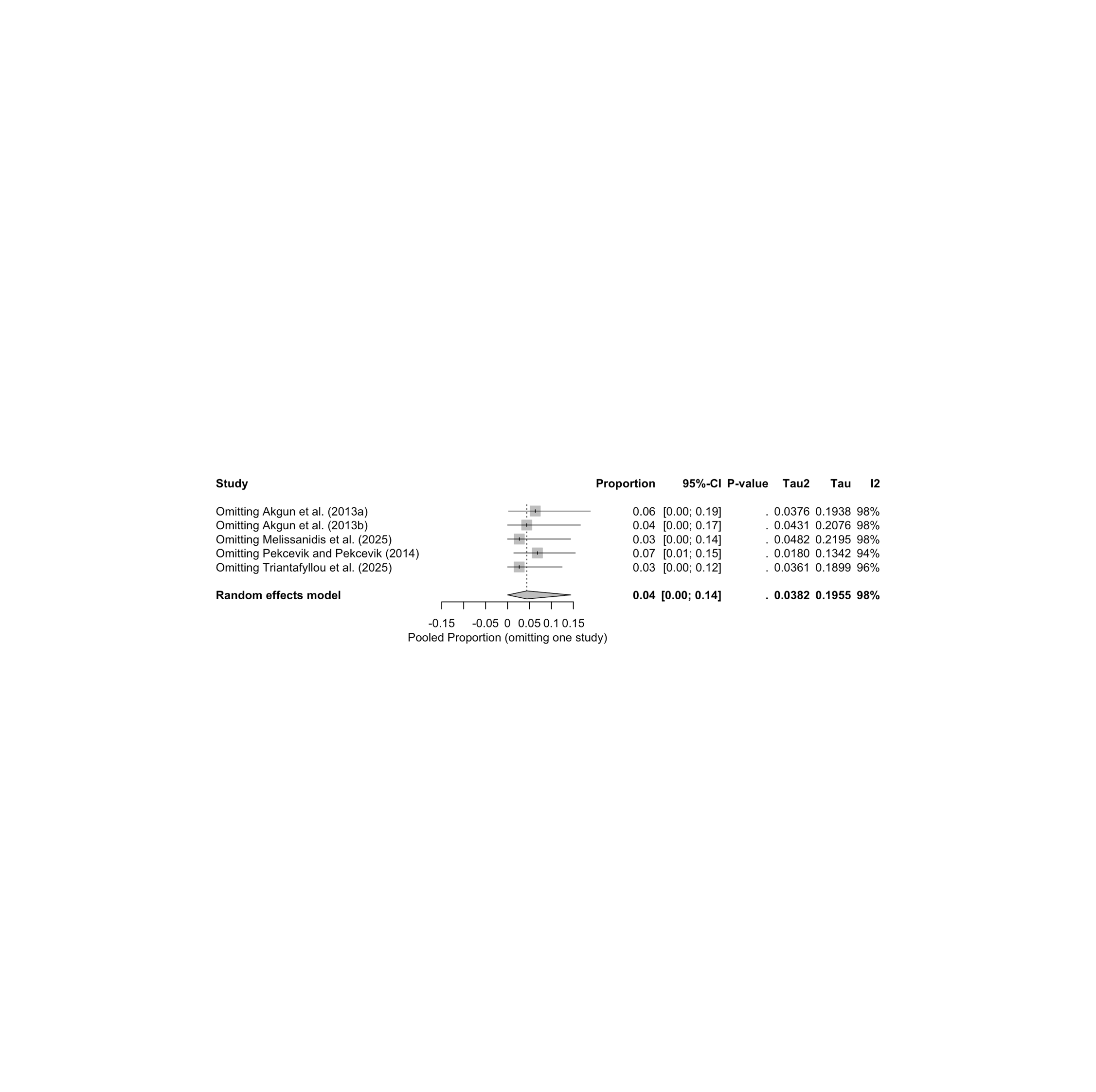
**

***Supplementary Figure 1.*** *Leave-one-out analysis for the aberrant posterior inferior cerebellar artery pooled prevalence estimate.*

**
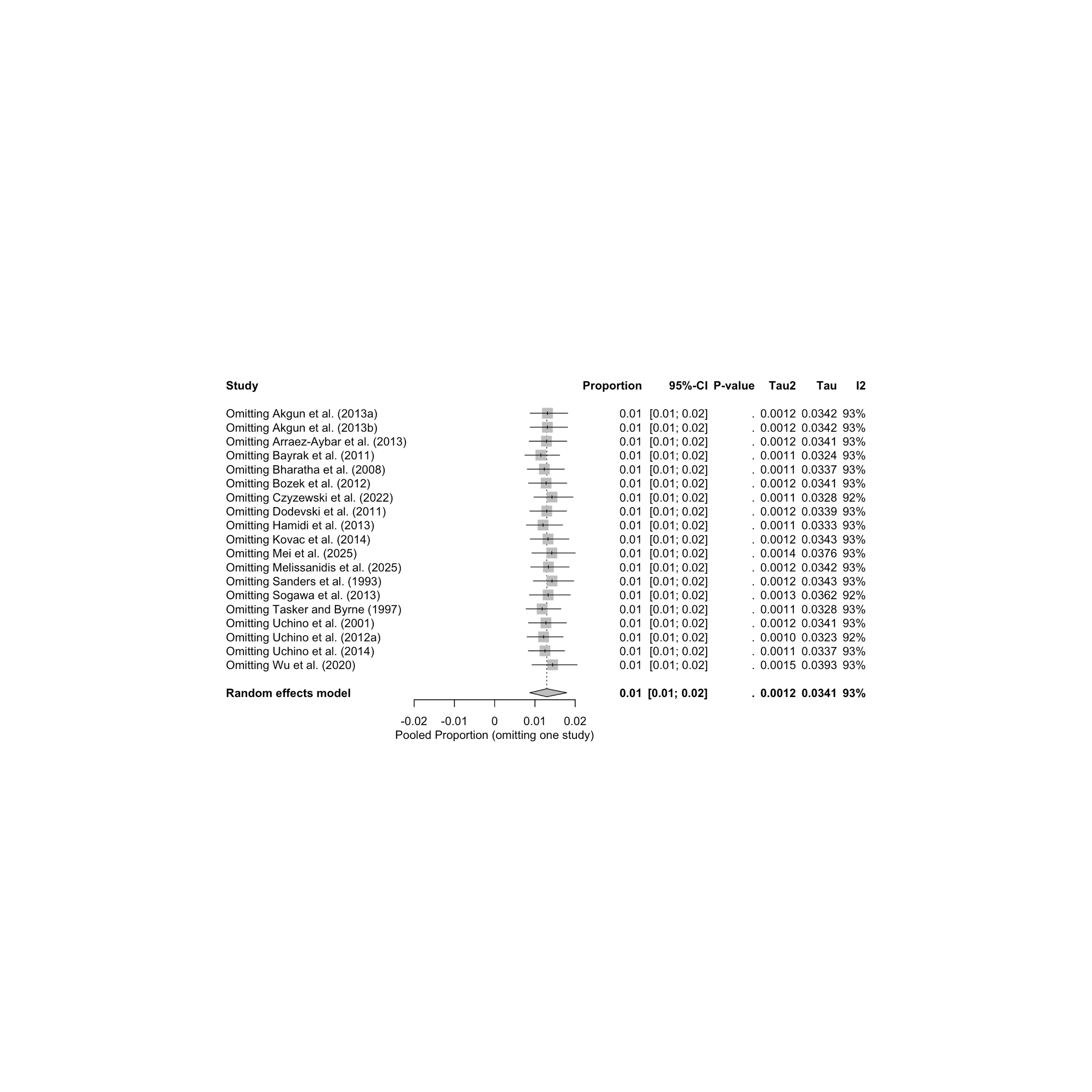
**

***Supplementary Figure 2.*** *Leave-one-out analysis for the basilar artery fenestration pooled prevalence estimate.*

**
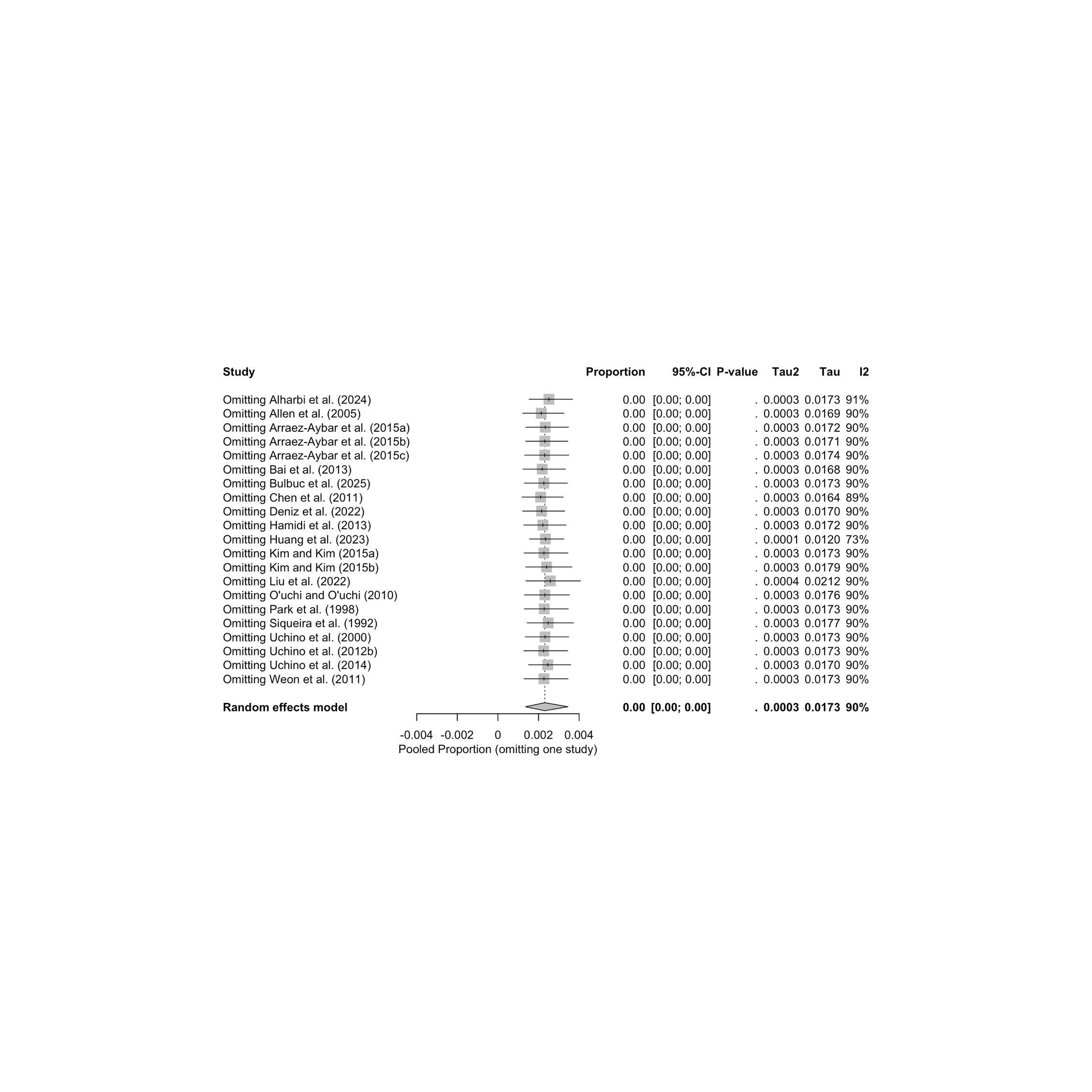
**

***Supplementary Figure 3.*** *Leave-one-out analysis for the persistent trigeminal artery pooled prevalence estimate.*
